# Physiological and Transcriptomic Changes during the Early Phases of Adventitious Root Formation in Mulberry Stem Hardwood Cuttings

**DOI:** 10.3390/ijms20153707

**Published:** 2019-07-29

**Authors:** Chunqiong Shang, Honglei Yang, Sang Ma, Qiudi Shen, Li Liu, Chengxiang Hou, Xu Cao, Jialing Cheng

**Affiliations:** 1 College of Biotechnology, Jiangsu University of Science and Technology, Zhenjiang 212003, China; 2 Key Laboratory of Silkworm and Mulberry Genetic Improvement, Ministry of Agriculture and Rural Areas, Sericultural Research Institute, Chinese Academy of Agricultural Sciences, Zhenjiang 212018, China

**Keywords:** cutting propagation, adventitious rooting, root primordia, phytohormone, sugar metabolism, RNA-seq, *Morus*

## Abstract

The initiation and induction of root primordia are of great importance for adventitious root (AR) formation in cutting propagation of horticultural and forestry crops. However, the underlying mechanisms orchestrating these early phases of AR formation remain largely unexplored. Here, we investigated the physiological and transcriptomic changes during the early AR phases in mulberry stem hardwood cuttings. The results showed that the concentrations of soluble proteins increased, whereas concentrations of soluble sugars and starch were decreased. Indole-3-acetic acid (IAA) and zeatin had a rapid transit peak at 6 h after planting (hAP) and declined thereafter. The activities of peroxidase and catalase persistently increased and indole-3-acetic acid oxidase was maintained at a higher stable level from 0 hAP, while the activities of polyphenol oxidase fluctuated with soluble phenolics and IAA levels. The comparative transcriptome identified 4276 common genes that were differentially regulated at −6, 0 and 54 hAP. They were separated into five clusters with distinct biological functions such as defense response and photosynthesis. Considerable common genes were assigned to pathways of sugar metabolism, mitogen-activated protein kinase, and circadian rhythm. The gene co-expression network analysis revealed three major co-expressed modules involved in stress responses, hormone signaling, energy metabolism, starch metabolism, and circadian rhythm. These findings demonstrate the positive effect of auxin on AR induction, and uncovered the crucial roles of stress responses, hormone signaling and circadian rhythm in coordinating the physiological changes during the early phases of AR formation in mulberry stem hardwood cuttings.

## 1. Introduction

Vegetative propagation is an ideal method for reproducing plants in vitro. It largely accelerates the propagation cycle while preventing genetic segregation due to variable seed production [1]. Adventitious root (AR) development is the key step for successful vegetative propagation (except grafting). Tissues of origin are most frequently the pre-committed cells in cambium or adjacent vascular tissues in non-root organs such as leaves, stems and internodes [2]. AR formation may occur naturally or under exogenous environmental stimulus (e.g., wounding), which are characterized as different AR types presenting unique developmental physiologies [3]. Cutting propagation, one of the vegetative propagation techniques, is widely used in ornamental horticulture [4] and forestry [5,6] for large-scale reproduction of elite genotypes. The capacity to form ARs on stem cuttings is not universal, but instead is highly variable between and within plant species or even genotypes, and economic losses could be caused due to insufficient rooting ability or poor quality of the newly established root system [7]. AR formation in cutting propagation represents an excision-induced rooting type, which is a combined outcome of wounding at the cut site and isolation from the resource and signal network of the whole plant [2].

AR formation is controlled by genetic and environmental factors, among which the phytohormone auxin plays a central role [8]. Exogenous application of auxin (e.g., indole-3-acetic acid, IAA) can increase AR formation in cuttings of most plant species [9,10]. Cytokinins are also important regulators of AR formation in stem cuttings, but its relationship with AR formation is inconsistent [11,12]. Other hormones, such as gibberellic acid (GA) and jasmonic acid (JA), have been demonstrated to have a phase-dependent effect, being inhibitory in early induction but stimulatory in root formation [13,14]. In addition to the shift of phytohormone homeostasis, it was recently found that hormone functions involve a cross-talk with upstream transcription factors (TFs) [15], miRNA [16], and downstream metabolic signals such as reactive oxygen species (ROS) [17], some secondary metabolites such as flavonoids and phenolics [18], and carbohydrates [19] in several plant species such as black walnut, apple, wheat and petunia. The *WOUND INUCED DIFFERENTIATION* (*WIND*) genes, encoding the *APETALA2/ETHYLENE-RESPONSIVE ELEMENT BINDING FACTOR* (*AP2/EREBP*) family members, have been suggested to be key regulators of cell proliferation through activating cytokinin responses, but their mechanistic link with cell cycle remains to be addressed [20]. Genome-wide transcriptome analysis in the leaf explants of *Arabidopsis* identified several auxin-independent *NAC DOMAIN CONTAINING PROTEIN* (*NAC*) family members that might be associated with JA response for root regeneration [21]. Recent studies also addressed the auxin-induced upregulation of a TF network, including *AUXIN RESPONSE FACTOR* (*ARF*), *WUSCHEL RELATED HOMEOBOX* (*WOX*11) and *LATERAL ORGAN BOUNDARIES DOMAIN* (*LBD*16 and *LBD*29) required for formative cell division in AR formation [22,23,24]. Moreover, miRNAs can differentially regulate root development via their target genes. It is reported that a complex regulatory network of miR160/167 and three members of the family (*ARF*6, *ARF*8, and *ARF*17) played an important role in the fine-tuning of AR initiation in *Arabidopsis* [25]. However, the knowledge concerning the molecular mechanisms on AR formation is primarily based on systems of intact hypocotyls of *Arabidopsis* and lateral root development [26,27,28] because the signaling modulators between the initiation process of lateral and adventitious roots partially overlap [29]. Nevertheless, direct experimental evidence of AR formation in stem cuttings of non-model perennial woody trees is still scarce, and it is not clear whether these regulators serve similar functions in woody plant systems.

Mulberry (*Morus*) is an economically important tree species in sericulture industry whose leaves are the sole food source for silkworm (*Bombyx mori*). Since mulberry is highly heterozygous and characterized by a long juvenile period, saplings are propagated by vegetative means such as tissue culture, stem cuttings, bud or pocket grafting for commercial cultivation [30]. However, the use of cutting propagation at industrial levels is greatly hampered because of the poor ability to form ARs via both hardwood and leafy cuttings. To overcome the recalcitrance of AR formation, an improved cutting propagation technique suitable for most mulberry species has been established by our group, and the cuttings are rooted up to ca. 90% [31]. To better understand the physiological and molecular mechanisms underlying AR formation, and exploit the possible candidate genes that might be responsible for this process, a series of comprehensive works has been undertaken by our group in recent years [32,33,34,35,36]. The anatomical study reveals that AR formation was originated directly from the parenchyma cells neighbor to the cambial tissues instead of forming a callus at the cut site [37]. Furthermore, three developmental stages presenting distinct morphological changes were characterized: Stage 1 of cutting excision with a new cut site; stage 2 of stem base with dome-shaped expansion; and stage 3 with AR outbreaking the cortex [33]. By using transcriptome and proteome approaches, we found that stage 2 displayed stronger responses both in hardwood or leafy stems, suggesting the importance of the early phases in AR formation [33,35]. 

AR formation proceeds in multiple phases that require root founder cells to respecify cell fate and to develop into a new functional root system [7]. This process can be divided into three widely recognized phases: (1) Induction, reprogramming of pre-committed cells devoid of any visible cell divisions; (2) initiation, when cells start to divide and root primordia are formed; and (3) expression, involving the establishment of new vascular connection and root emergence from the stem [38,39,40]. Based on this concept, it is inferred that the expanding of the basal region of mulberry stem cuttings should mirror the early steps (induction and initiation) of AR formation, which occur within three to five days after planting [32,33]. A high quantity of root primordia is the prerequisite for AR formation; nevertheless, few studies have elucidated how AR formation is regulated at the early phases in woody crops, including mulberry [41,42]. Our previous studies focused on the biological events of the whole rooting process, while the transcriptomic reprogramming governing root competence preparations at the early AR phases remain unexplored. 

To address this important issue, here we present a detailed physiological and transcriptomic analysis of the first 60 h of AR formation in the basal region of mulberry stem hardwood cuttings. Specifically, the concentrations of soluble sugars, starch, soluble proteins, soluble phenolics, hydrogen peroxide (H_2_O_2_), superoxide anion radical (O_2_^•−^) were analyzed. Rooting-related enzymes including peroxidase (POD), catalase (CAT), indole-3-acetic acid oxidase (IAAO) and polyphenol oxidase (PPO), together with the phytohormones IAA and zeatin (ZA), were also determined. Furthermore, the transcriptomic profiles were investigated to uncover the key molecular players regulating early AR formation. This study can provide insights into the physiological and the molecular mechanisms underlying the early steps of AR formation in mulberry and other tree species reproduced via cutting propagation. 

## 2. Results

### 2.1. Physiological Changes during Early AR Phases

**Figure 1:**
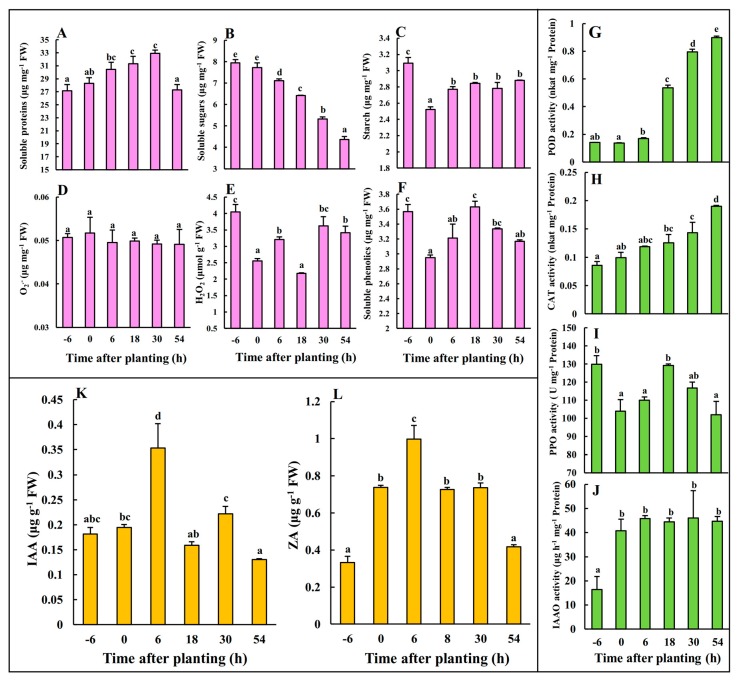
The dynamic changes of the concentrations of rooting-related metabolites including soluble proteins (**A**), soluble sugars (**B**), starch (**C**), O_2_^•−^ (**D**), H_2_O_2_ (**E**) and soluble phenolics (**F**), enzyme activities of POD (**G**), CAT (**H**), PPO (**I**) and IAAO (**J**), and phytohormones IAA (**K**) and ZA (**L**) during the early phases of adventitious root (AR) formation in mulberry stem hardwood cuttings. Mean ± SE are presented (*n* = 3). The different letters on the error bars indicate significant differences at *p* < 0.05.

Significant physiological changes related to protein and sugar metabolism, ROS and phytohormones occurred during the 54 h after planting (hAP), as shown in Figure 1. For instance, the concentrations of soluble proteins were increased to peak at 30 hAP, but dramatically decreased at 54 hAP (Figure 1A). The concentrations of soluble sugars were significantly decreased from 6 hAP until 54 hAP (Figure 1B), whereas starch was significantly reduced at 0 hAP and remained unchanged afterwards (Figure 1C). No differences were observed for O_2_^•−^ concentrations along the 54 hAP (Figure 1D). The concentrations of H_2_O_2_ and soluble phenolics were decreased at 0 hAP but displayed varied pattern afterwards (Figure 1E,F). The activities of POD and CAT were dramatically promoted from 0 to 54 hAP (Figure 1G,H). However, the activities of PPO were dynamically changed along 54 hAP (Figure 1I). The activities of IAAO were significantly increased at 0 hAP, and this level was maintained to 54 hAP (Figure 1J). The concentrations of IAA and ZA were significantly induced and reached to peak at 6 hAP, but generally declined afterwards (Figure 1K,L). 

### 2.2. Comparative Transcriptomes Uncover Sets of Important Common Genes during Early AR Phases

**Figure 2:**
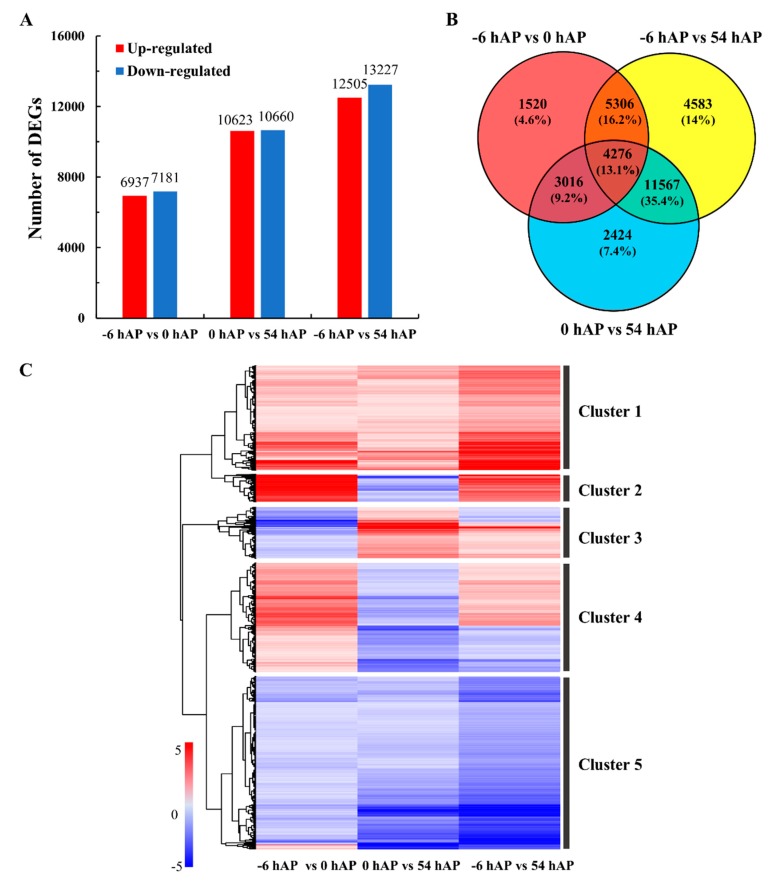
Differentially expressed genes (DEGs) from RNA-seq during the early phases of AR formation in mulberry stem hardwood cuttings. The number of DEGs in the three time point comparison groups (−6 hAP vs. 0 hAP, 0 hAP vs. 54 hAP, −6 hAP vs. 54 hAP) (**A**), the Venn diagram presentation of the overlapped DEGs (**B**), and the hierarchical clustering analysis of the 4276 common DEGs based on data of log_2_Ratio for each gene (**C**), are presented. The common genes are divided into five clusters. The color bar, ranging from blue to red, indicates low to high changes of transcriptional expression level from −5 to 5.

Taking the advantages of RNA-seq, the global gene reprogramming profiles at −6, 0 and 54 hAP were monitored, and the differentially expressed genes (DEGs) in each comparison group were obtained (Table S1 and Figure 2A). Correlation analysis found high similarities in samples of the same time point, while the samples from −6 hAP were much closer to those from 0 hAP than from 54 hAP (Figure S1). There were 6937 and 7181 genes that were up- and down-regulated in −6 hAP vs. 0 hAP, respectively (Figure 2A). More genes were differentially expressed in 0 hAP vs. 54 hAP (10,623 up-regulated and 10,660 down-regulated, respectively) than −6 hAP vs. 0 hAP (Figure 2A). The numbers of DEGs were highest in −6 hAP vs. 54 hAP among all the three comparisons (Figure 2A). The three comparison groups shared 4276 DEGs which accounted for ca. 13% of all the detected DEGs, and hence termed common genes (Table S2 and Figure 2B). The accuracy of RNA-seq was validated by RT-qPCR, and a high correlation between the results obtained from RNA-seq and RT-qPCR was observed (Figure S2).

The common DEGs can be clearly divided into five clusters with distinct gene expression profiles by hierarchical clustering (Figure 2C). For instance, the genes assigned to Cluster 1 were up-regulated in the three comparison groups, and enriched in the important pathways related to plant-type cell wall (Table S3 and Figure 2C). Cluster 2 represented the genes involved in defense and stress responses, which were up-regulated in −6 hAP vs. 0 hAP and −6 hAP vs. 54 hAP, and down-regulated in 0 hAP vs. 54 hAP (Table S3 and Figure 2C). Genes assigned to Clusters 3 and 4 displayed relatively diverse expression profiles, and no significant GO or KEGG pathways were enriched in these two clusters (Table S3 and Figure 2C). For Cluster 5, almost all genes (expect a very small proportion) were inhibited in the three comparison groups, and they participated in vital pathways related to photosynthesis, energy metabolism, stress responses, and response to pathogen attack (Table S3 and Figure 2C). The photosynthetic components were significantly modified according to the cellular component results of Cluster 5 (Table S3). Notably, several clues suggested that the translation processes were modified, since RNA metabolism, the mRNA/RNA catabolic process, and the pyridine nucleotide metabolic process were enriched in the genes that belonged to Cluster 5 (Table S3).

### 2.3. Several Pathways were Significantly Modified during Early AR Phases

**Figure 3:**
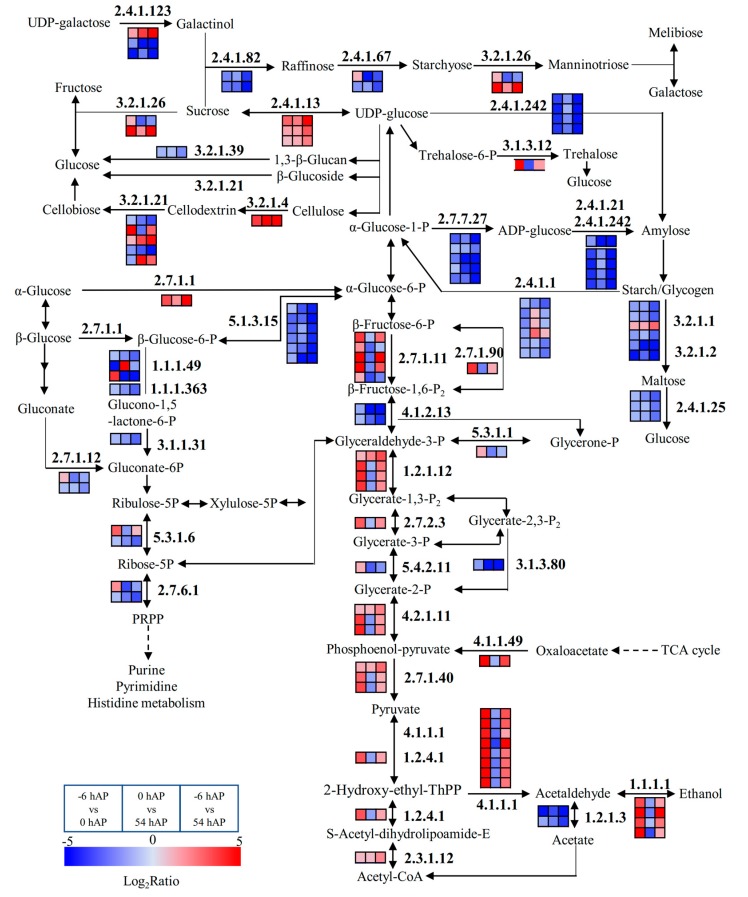
The significantly expressed common genes involved in starch and sucrose metabolism, glycolysis, pentose phosphate pathway and galactose metabolism during the early phases of AR formation in mulberry stem hardwood cuttings. The enzyme numbers of the enriched genes are in bold, and the changes of transcriptional expression levels in the three comparison groups are illustrated with colored boxes. The boxes from left to right indicate the expression values obtained in −6 hAP vs. 0 hAP, 0 hAP vs. 54 hAP, and −6 hAP vs. 54 hAP, respectively. Each row of boxes represents a mulberry homolog gene. The color code from blue to red represents the changes of transcriptional expression level between –5 and 5. This figure is redrawn based on the KEGG pathways.

The pathways involved in sugar synthesis metabolism were globally inhibited, these soluble sugars included galactose, glucose, fructose, except sucrose (Table S4 and Figure 3). Three genes encoding *SUCROSE SYNTHASE* (*SUS*) were significantly up-regulated in these three comparison groups (Table S4 and Figure 3). Additionally, the genes involved in pentose phosphate pathway were significant down-regulated, implying the attenuated capacities of synthesis of 5-phosphate ribose that can be used for ribose synthesis (Table S4 and Figure 3). In contrast, the catabolism of glucose into energy via glycolysis was generally enhanced, but displayed higher responsiveness in −6 hAP vs. 0 hAP (Table S4 and Figure 3). 

**Figure 4:**
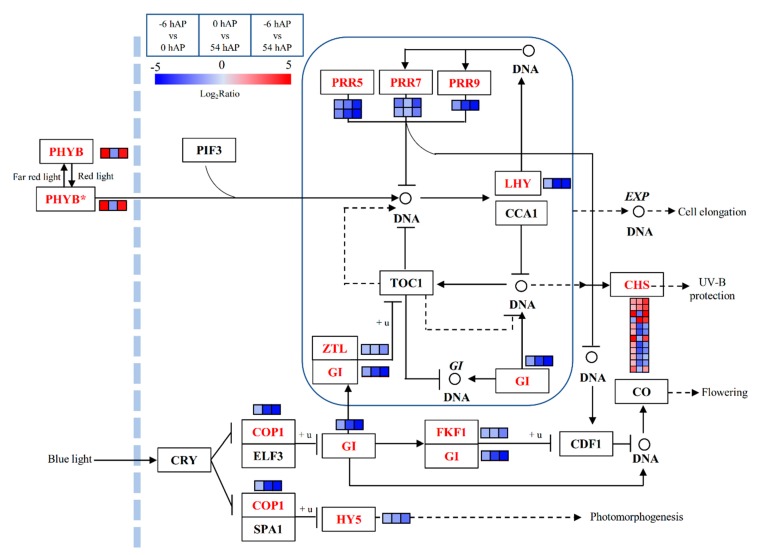
The significantly expressed common genes involved in the circadian rhythm plant pathway during the early phases of AR formation in mulberry stem hardwood cuttings. The enriched common genes are in red, and the changes of transcriptional expression levels in the three comparison groups are illustrated with colored boxes. The boxes from the left to right indicate −6 hAP vs. 0 hAP, 0 hAP vs. 54 hAP, and −6 hAP vs. 54 hAP, respectively. Each row of boxes represents a mulberry homolog gene. The color code from blue to red represents the changes of transcriptional expression level between –5 and 5. The solid or dashed arrows indicate direct or indirect positive effects, and the T type lines indicate inhibitory effects. This figure is redrawn based on the KEGG pathways.

The genes participating in the circadian rhythm plant pathway were globally inhibited, with some exceptions (Table S4 and Figure 4). For instance, the input element that senses light quality (i.e., *PHYTOCHROME B*—*PHYB*), was up-regulated in −6 hAP vs. 0 hAP and −6 hAP vs. 54 hAP, and down-regulated in 0 hAP vs. 54 hAP (Table S4 and Figure 4). Several genes encoding *CHALCONE SYNTHASE* (*CHS*) involved in UV-B protection were differentially regulated in these three comparison groups (Table S4 and Figure 4), suggesting the diverse regulatory functions played by *CHS* in response to light signals. The remaining significant genes that assigned to this pathway were consistently inhibited, including *PSEUDO-RESPONSE REGULATOR 5* (*PRR5*), *PRR7*, *PRR9*, *GIGANTEA* (*GI*) and *ELONGATED HYPOCOTYL 5* (*HY5*) (Table S4 and Figure 4).

**Figure 5:**
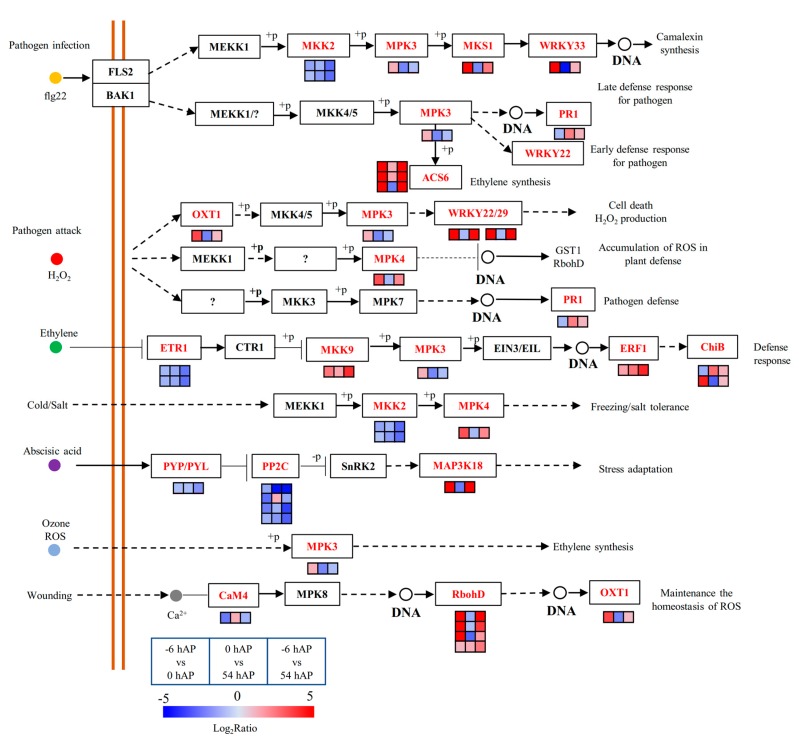
The significantly expressed common genes assigned into the MAPK signaling pathway during the early phases of AR formation in mulberry stem hardwood cuttings. The enriched common genes are in red, and the changes of transcriptional expression levels in the three comparison groups are illustrated with colored boxes. The boxes from the left to right indicate −6 hAP vs. 0 hAP, 0 hAP vs. 54 hAP, and −6 hAP vs. 54 hAP, respectively. Each row of boxes represents a mulberry homolog gene. The color code from blue to red represents the changes of transcriptional expression level between –5 and 5. The solid or dashed arrows indicate direct or indirect positive effects, and the T type lines indicate inhibitory effects. This figure is redrawn based on the KEGG pathways.

The mitogen-activated protein kinase (MAPK) signaling pathway integrates multiple signals such as H_2_O_2_, NO, cGMP, and Ca^2+^, which bridges the links between H_2_O_2_ and auxin to facilitate AR formation in plants [43]. Several genes involved in ethylene synthesis and signaling were significantly promoted in these three comparison groups, such as *1-AMINOCYCLOPROPANE-1-CARBOXYLIC ACID SYNTHASE 6* (*ACS6*) and *ETHYLENE RESPONSE FACTOR* (*ERF*) such as *ERF1*, *ERF*3, *ERF5*, *ERF*6, *ERF*110 and *ERF*115 (Table S4 and Figure 5). One gene encoding the *PATHOGENESIS-RELATED GENE 1* (*PR1*), the molecular marker for systemic acquired resistance (SAR) which is highly SA responsive, was upregulated under the latter two comparison groups (Table S4 and Figure 5). Besides, several genes belonging to the *BASIC CHITINASE* (*CHiB*) gene family were also globally up-regulated (Table S4 and Figure 5).

### 2.4. TFs and Hormone-Related Genes Play Essential Roles during Early AR Phases

In total, 244 genes (ca. 6% of the 4276 common genes) were identified as TFs that belonged to 45 gene families (Table S5). *BASIC HELIX-LOOP-HELIX* (*bHLH*), *AP2/EREBP*, *MYB DOMAIN PROTEIN* (*MYB*) and *WRKY DNA-BINDING PROTEIN* (*WRKY*) represented the most abundant gene family (Table S5). Some of these TFs were involved in the biosynthesis or signaling pathways of hormones like ethylene, auxin, cytokinin and GA (Table S5). A total of 247 genes were identified to be involved in diverse hormone-related processes, such as biosynthesis, metabolism, transport and perception (Table S6). For instance, 18 homologs encoding auxin efflux carriers *ATP-BINDING CASSETTE B* (*ABCB*); and 8 homologs encoding *PIN-FORMED* (*PIN*), including *PIN1*, *PIN3*, *PIN4* and *PIN6*, were globally promoted in the three comparison groups (Table S6). These data indicate the important roles played by TFs and hormones in early AR phases.

### 2.5. The Common DEGs were Highly Co-Expressed

**Figure 6:**
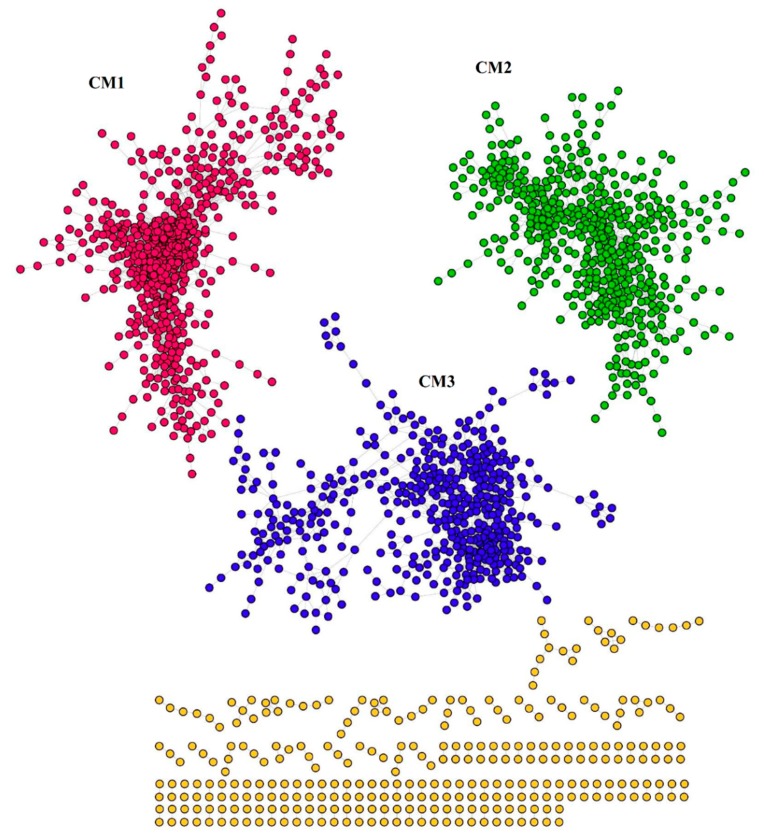
The co-expression network of the 4276 common DEGs in the three different time point comparison groups during the early phases of AR formation in mulberry stem hardwood cuttings. Three major co-expression modules (CMs, subnetwork with more than 30 genes) were identified, and the clustered dots with different colors represent genes belonging to a specific CM, the scattered yellow dots indicate non-co-expressed genes.

Among the 4276 common genes, 1536 genes (nodes) were highly co-expressed via 9213 correlations (edges) (Figure 6). This co-expression network presented three co-expressed modules (CMs), that is, CM1, CM2 and CM3, with 604, 476, and 456 assigned genes, respectively (Table S2 and Figure 6). These CMs were enriched in distinct biological pathways (Table S7). For instance, CM1 was enriched in pathways involved in defense responses, such as wounding, fungus, SAR and immune response (Table S7). CM2 was mainly related to metabolism processes and rhythmic regulation, including starch metabolic and biosynthetic process, circadian rhythm, rhythmic process, cellular glucan metabolic process, as well as several other responses to light that could also attribute to rhythmic regulation (Table S7). Additionally, several pathways related to transcription activities were enriched in this CM, such as rRNA processing, mRNA modification and rRNA metabolic process (Table S7). As for CM3, pathways involved in cell wall modification, nutrition transport and defense responses were enriched in this CM (Table S7).

## 3. Discussion

Carbohydrates have been considered one of the key factors that contribute to AR formation. The depletion of carbohydrate, together with increasing energy metabolism, have been widely observed in many plant species [19,44,45,46]. The maintained high levels of energy via respiration are associated with (1) compensating for the energy loss from photosynthesis, (2) respiratory burst processes triggered by the defense responses, and (3) the energy and internode substrates required for AR formation [46,47]. In line with this, the decreased soluble sugars, together with the more abundant mRNA levels of several genes that participate in the tricarboxylic acid (TCA) cycle, highlight the important roles of sugar metabolism processes in the early phases of AR formation. In contrast, the inhibitory effects of transforming sucrose into other forms of sugars, such as galactose, glucose, fructose and ribose, were observed by the transcriptomic analysis, suggesting that the respiration pathways contributed to the decline in concentration of soluble sugars. Moreover, the increased soluble protein concentrations implied that the enhanced protein synthesis capacities may be maintained by the high levels of energy and substrates derived from respiration. Interestingly, in line with the accumulated protein levels, degradation of amino acids was inhibited, whereas synthesis pathways of amino acids were globally promoted, implying the links between nitrogen metabolism and root development [46,48]. This was also the case for protein degradation and synthesis. Both amino acid and proteins represent the most important nitrogen-containing compounds in plants [49,50]; thus, the changes in these pathways suggest a closely coordinated linkage between protein synthesis and sugar metabolism, which plays a crucial role in the early AR phases in mulberry. 

Auxin has been widely accepted as the master regulator of AR formation, whereas high auxin levels obviously have an inhibitory role at later stages [7]. Generally, the IAA concentration at stem base showed a gradual decrease, except for the transit peak detected at 6 hAP, along with the increased activities of IAAO and POD throughout the early phases of AR formation. Moreover, these physiological data correspond well to the repressed active IAA at transcriptional level, supporting a key concept that the endogenous IAA pool shifts toward long-term reduction for AR formation in cuttings [2]. Similar early accumulation of IAA has been detected in founder cells of lateral roots in *Arabidopsis* [51] and AR formation in other plant cuttings [52,53]. Our results verify that early auxin accumulation should be a universal event boosting AR formation in cuttings, whereas exogenous auxin (e.g., indole-3-butyric acid—IBA) might enhance the signal of auxin directing to founder cells for AR initiation [54,55]. Previous research demonstrates that excision-induced AR formation in cuttings is dependent on polar auxin transport (PAT) [56,57,58]. PAT relies on specific transporters for influx and efflux [59,60]. Coincidently, over half of the DEGs participating in auxin transport were rapidly up-regulated, especially a large proportion of the *ABCB* and *PIN* members, which are auxin efflux carriers involved in basipetal auxin transport and organogenesis [58]. Overexpression of *ABCB19* yielded more AR formation than the wild type in intact *Arabidopsis* hypocotyls, whereas *pin1* and *abcb19* mutants displayed a reduced number of ARs [57]. Notably, mulberry homologs of *ABCB11*, *ABCB*15, *ABCB*18 and *ABCB*22 were significantly stimulated at 0 hAP, whereas the other two members of ABC transporter (*ABCB19* and *ABCG36*) and eight homologs of *PIN* displayed the most profound induction at 54 hAP. These results support that *ABCB* and *PIN* genes might participate in mulberry AR initiation by promoting auxin lateral efflux from phloem toward vascular parenchyma, the root founder cells, to trigger subsequent auxin accumulation [61]. On the contrary, less auxin influx transporters were differentially expressed compared with the efflux carriers. These data further suggest a preferential role of auxin efflux carriers during early AR phases.

Similar to IAA changes, the cytokinin concentration was induced quickly and decreased to a steady-state level afterwards. However, genes involved in cytokinin degradation, transport and signaling displayed phase-specific expression patterns. It is well documented that exogenous application of cytokinin suppresses AR primordia formation by inhibiting the genes involved in biosynthesis, transport and signaling of auxin [62]. The homeostasis of auxin level was modulated by a feedback loop closely related to cytokinin [63]. It is anticipated that, in mulberry stem cuttings, cytokinin might be more involved in the expression phase of AR formation when vascular differentiation takes place, as previously reported [64]. Ethylene and JA are both wound-induced phytohormones. Our previous study has found increased transcriptional levels of genes encoding *ACS*, the rate-limiting enzyme in ethylene biosynthesis at the AR expression phase [33]. In this study, *ACS6* homologs were persistently up-regulated, except for *ACS9* and *ETHYLENE OVERPRODUCER 1* (*ETO1*), a negative regulator of *ACS*, which displayed up- and down-regulation until 54 hAP, indicating a phase-dependent differential activation of ethylene biosynthesis. In *Petunia* *hybrida* cuttings, a strong accumulation of JA was found during the induction phase of AR formation, suggesting that it possibly acts as an accelerator of AR formation [65]. In this study, however, a large proportion of genes (e.g., A*LLENE OXINE SYNTHASE*, *AOS*) involved in JA biosynthesis were up-regulated at 54 hAP. These data imply that ethylene might play a positive role in the induction and initiation phases of AR formation whereas JA is more strongly associated with the later initiation phase in mulberry cuttings.

TFs of the GRAS, AP2/EREBP and WOX family proteins are well known for linking auxin signaling with cell fate determination and specification, in addition to being involved in the feedback regulation of local auxin homeostasis [66]. *SCARECROW* (*SCR*) and *SHORTROOT* (*SHR*) are auxin inducible, and important for early root meristem patterning and maintenance. Among the identified *GRAS* genes, *SCARECROW*-LIKE 1 (*SCL1*) homologs were significantly induced in the early phases. It has been found that *SCL1* is specifically induced and located in the cambial zone and derivative cells involved in root meristem initiation and in the root primordium in rooting competent microshoots of *Castanea sativa* [67]. This cell-type and phase-dependent manner could also play an important role in mulberry early AR phases. In the current study, the *AP2/EREBP* family presented the most responsive common DEGs compared to the other families. Genes of this family showed a global up-regulation, which was closely associated with auxin and cytokinin signaling in root regeneration [68,69,70]. A good example is *RAP2.6L*, which was involved in pith cell division and displayed a great induction in response to the reduced auxin level at the lower side of incision in *Arabidopsis* stems [71]. This is in line with our data and these TFs might also be at work for cell division in the organization of root primordia. Previous literature confirms the role of several *WOX* genes in promoting meristem identity during wounding-induced AR formation [22]. *WOX7* and *WOX13* homologs were found up-regulated immediately after excision in this study. In *Arabidopsis*, both WOX7 and WOX13 proteins are key transcriptional regulators implicated in the primary and/or lateral root initiation and development [72]. Functional analysis suggests that WOX7 protein regulates lateral root development through direct repression of cell cycle genes, particularly *CYCLIN D 6* (*CYCD6*), and it plays an important role in coupling the lateral root development program and sugar status in plants [73]. 

Many previous studies have documented that genes involved in similar pathway tend to be co-expressed [74,75]. A transcriptomic regulatory network was constructed to illustrate the coordinated relationships of the genome-wide gene expression profiles underlying the early stages of AR formation in mulberry. GO analysis revealed three CMs with several specifically overlapping pathways, suggesting that these CMs might be finely regulated by their common pathways. For instance, the CM1 represented the defense response module dealing with wounding caused by cutting. Pathways involved in plant immune system were significantly over-represented in this CM, such as SAR, and regulation of immune response. In line with the above-mentioned aspects regarding the decreased soluble sugars, the enriched pathways involved in respiratory burst in CM1 strongly supported the conclusions drawn in the upper paragraph. Additionally, several clues also demonstrate the stress phytohormones, such as JA, SA, and ethylene, were deeply involved in these defense responsive reactions during the early stages of AR formation in mulberry, which is the same for many other plants in the same biological processes [2,46]. 

In addition to CM1, CM2 was obviously related to starch and circadian rhythm. The pathways involved in starch metabolism and biosynthesis were significantly enriched in CM2. The reserved starch in the cuttings is thought as the carbon source for AR formation because photosynthetic assimilation is impossible [46]. It is also well documented that starch degrades earlier than sugars during the AR process [19,44,45,46], which stays in line with our results. Correspondingly, the soluble sugars drastically declined from 6 hAP; otherwise, the starch declined at 0 hAP and remained unchanged during the whole process. The explanation for this might be the differences in the availability of sugar and starch, as the former one can easily subject to glycolysis and TCA pathways for energy. The role of circadian rhythm in AR formation in mulberry was elucidated recently by our group, which showed it can coordinate the signaling of phytohormones such as ethylene and auxin, and promote root primordium induction [33]. Here, we showed again that the circadian rhythm also participated in early AR phases. Moreover, the relationship between the circadian rhythm and starch have been demonstrated in many plants and biological processes [76,77,78]. Thus, the co-enrichments of circadian rhythm and starch metabolism in CM2 imply the close links between these two processes. However, the transcriptional changes in genes involved in starch metabolism did not modify the concentrations of starch, suggesting that some other regulatory layers or compensation pathways independent of circadian rhythm might be existed, which needs to be further explored. 

In conclusion, dramatic physiological and transcriptomic changes occurred during the initiation and induction phases of AR formation in mulberry stem hardwood cuttings. The physiological profiles of soluble proteins, soluble sugars and starch were in good accordance with the enriched pathways of protein synthesis, glycolysis, TCA cycle from the transcriptomic data. The transit IAA maxima and the activated expressions of auxin efflux transporters at stem base indicated its importance as one of the first input signals for root cell competence preparation. On the other hand, several sets of DEGs involved in pathways of sugar metabolism, circadian rhythm and MAPK were identified. A gene co-expression analysis based on the common genes revealed three highly co-expressed CMs enriched in distinct biological events, including stress responses, hormone signaling, energy metabolisms, photosynthesis and circadian rhythm. These findings suggest the positive effect of auxin on AR induction, and have uncovered the crucial roles of hormone signaling, stress responses and circadian rhythm in coordinating the physiological changes during the early stages of AR formation in mulberry stem cuttings.

## 4. Materials and Methods

### 4.1. Cutting Cultivation and Harvest

Healthy and dormant branches were collected from mulberry cultivar YU711 conserved at the mulberry germplasm nursery of the Sericulture Research Institute, Chinese Academy of Agricultural Sciences (Zhenjiang, China). The preparation progress of cuttings was as previously described [33]. Briefly, the branches were cut into cuttings with uniform size (ca. 12 cm in length) and treated with 1000 mg/L carbendazin to avoid mildew and decay. Subsequently, the basal 3 cm of the cuttings were dipped for 8–10 s in 200 mg/L IBA dissolved in 50% ethanol and planted in a moist perlite medium. Cuttings were cultivated at 28 °C and watered regularly to keep humidity. During the rooting process, basal cortex (1–1.5 cm) of the cuttings were collected at 0, 6, 18, 30, 54 h after planting (hAP). Cuttings not treated with rooting solution were set as the control (−6 hAP). All the bark samples were partitioned into 0.5 cm segments and stored at −80 °C following fast-frozen in liquid nitrogen. For each time point, bark tissues from 15 cuttings were scraped and pooled as one replicate, three biological replicates were prepared. 

### 4.2. Analysis of Rooting-Related Metabolites

The concentrations of total soluble sugars and starch were determined using the anthrone-sulphuric acid method as suggested [79]. The fine powder of bark samples (ca. 100 mg) was extracted in 80% ethanol for 30 min at 80 °C and then centrifuged at 6000× *g* for 10 min. After the collection of the first supernatant, the pellet was extracted again as mentioned above, the supernatant was combined with the first one. Then, 2 mL of the supernatant was gently mixed with 5 mL of anthrone-sulphuric acid reagent and fully reacted in a boiling water bath for 10 min. The concentration of total soluble sugars was calculated spectrophotometrically by recording the absorbance at 620 nm after the reaction was cooled down. To analyze starch content, the pellets retained after the extraction of the soluble sugars were gelatinized in a boiling water bath for 15 min, and were extracted twice in 0.5 mL of 9.2 M and 4.6 M HClO_4_, respectively. The starch concentration in the supernatant was determined as that for soluble sugars. A standard curve was generated by using a serial of diluted solutions of glucose and the starch concentrations were expressed as glucose equivalents.

Soluble phenolics were determined as reported previously [80]. The frozen fine powder (ca. 60 mg) was extracted in 1.5 mL of 50% methanol and incubated in an ultrasonic bath for 1 h at 40 °C. After centrifugation (4500× *g*, 4 °C, 10 min), the supernatant was collected. The pellet was extracted again as mentioned above and the supernatant was collected and combined with the previous one. Subsequently, 1.5 mL of Folin-Ciocalteus reagent was added to 300 μL of the supernatant the reaction solution was kept in dark for 3 min at room temperature. Then, 1.2 mL of 7.5% Na_2_CO_3_ (*w*:*v*) was added and the mixture was incubated for 30 min in dark at RT. The absorbance at 765 nm of the mixture was recorded to determine the soluble phenolics content. Standard curve was prepared using a serial of diluted solutions of catechin (Sigma, St Louis, MO, USA).

The concentrations of H_2_O_2_ and O_2_^•−^ in plant materials was analyzed after [81]. The fine powder of fresh tissues (ca. 60 mg) was homogenized in 2 mL cold cetocaustin (5%) and centrifuged (10,000× *g*, 4 °C, 10 min). The supernatant was collected. After adding 0.1 mL of 20% TiCl4 in hydrochloric acid and 0.2 mL of 25% aqueous ammonia to 1 mL of the supernatant, the mixture was immediately centrifuged again (6000× *g*, 4 °C, 15 min). Subsequently, the supernatant was discarded and the pellet was dissolved in 3 mL of 1 M H_2_SO_4_. The absorbance was recorded spectrophotometrically at 410 nm. The concentration of O_2_^•−^ was determined spectrophotometrically at 530 nm, as described previously [81].

### 4.3. Assay of Rooting-Related Enzyme Activities

The enzyme activities of POD (EC 1.11.1.7) and CAT (EC 1.11.1.6) were determined spectrophotometrically by recording the increase/decrease of absorbance at 470 and 240 nm, respectively [82]. PPO (EC 1.14.18.1) activity was assayed according to [83] with some modifications. In brief, 1 mL of 100 mM 4-methylcatechol and 100 μL of the protein extract were submitted to 2 mL of 50 mM sodium phosphate buffer (pH 7.0) and preheated at 40 °C. Absorbances at 410 nm were recorded for 3 min. One unit of PPO activity was defined as the amount of enzyme that caused an increase in absorbance of 0.01 per minute. Results are expressed as units per milligram of protein (U mg^−1^ protein). IAAO (EC 1.10.3.3) activity was determined spectrophotometrically following previously reported methods [84], with minor modifications. Briefly, the reaction mixture containing 0.1 mM MnCl_2_, 0.1 mM 2,4-dichlorophenol, 40 μg/mL IAA, 7 mM phosphate buffer (pH 6.0) and 0.25 mL enzyme extract was incubated at 30 °C for 15 min. Then 1 mL of the reaction solution was mixed with 2 mL of the chromogenic reagent (0.5 M FeCl_3_: 35% perchloric acid = 1:50, *v*/*v*) and further incubated at 30 °C for 30 min in dark. The absorbance was measured at 530 nm. The IAAO activity was expressed as micrograms IAA destroyed per milligram of protein per hour (μg IAA mg^−1^ protein h^−1^). To quantify the activities of rooting-related enzymes on the protein basis, soluble proteins were extracted from 200 mg frozen homogenized samples in 2 mL of 50 mM phosphate buffer (pH 7.0). After centrifugation (10,000× *g*, 4 °C, 10 min), the supernatants were reacted with Coomassie brilliant blue G-250 for 5 min, and the absorbances were recorded at 595 nm. Standard curves were established using bovine serum albumin (BSA, Sangon Biotech, Shanghai, China) [85].

### 4.4. Quantification of Phytohormones

IAA and ZA were extracted following previous procedures [86,87] with some modifications [88]. Frozen homogenized samples (ca. 150 mg) were suspended in 1 mL of a cold extraction reagent (methanol: distilled water: acetic acid = 80: 20: 1, *v*/*v*/*v*). The mixture was subsequently shaken at 4 °C overnight before centrifugation for 10 min at 8000× *g*. The supernatant was collected and the debris was extracted again in 0.5 mL of the extraction reagent for 2 h. The supernatants were combined after each extraction and fully dried at 40 °C under N_2_ until the organic phase was completely removed. Subsequently, the residues were submitted to 0.5 mL of petroleum ether 3 times for decolorization at 60–90 °C and the organic phase was discarded. For ZA measurement, the lower layer was extracted three times with an equal volume of ethyl acetate after adjusting to an alkaline pH level before dried under N_2_. For IAA measurement, the aqueous phase was directly dried at 60 °C under N_2_, and resuspended in 0.5 mL of mobile phase with vigorous vibration. The phytohormone concentrations in the samples were analyzed using a high-performance liquid chromatography system (Agilent 1100 Series HPLC Value System, Waldbronn, Germany). A total of 10 μL of filtrated samples was separated within a reversed phase C_18_ column (250 mm × 4.6 mm, 5 μm, Kromasil). The operating conditions were a mobile phase of methanol: water (3:7, *v*/*v*) for ZA and methanol: water: acetic acid (400:600:6, *v*/*v*/*v*) for IAA, a flow rate of 0.8 mL/min and a temperature of 35 °C. Under these conditions, the retention time was 11.2 and 16.5 min for ZA and IAA, respectively. IAA and ZA were purchased for the preparation of standard curves to quantify hormone concentrations in the samples.

### 4.5. Library Preparation and Sequencing

To uncover the transcriptomic changes during the early AR phases, bark samples from the three time points, that is, −6 hAP, 0 hAP, and 54 hAP, were selected for transcriptomic analysis. Total RNAs were isolated using RNA Plus Reagent (Takara, Japan) following the manufacturer’s instructions. For each time point, three biological replicates were used. The integrity of RNA samples was verified using an Agilent 2100 BioAnalyzer (Agilent, Santa Clara, CA, USA). To produce reference sequences, equal amounts of the quantified RNA samples from the three time points were pooled for de novo RNA transcriptome analysis with Illumina Hiseq 2500 system (Illumina, San Diego, CA, USA) by synthesis with paired-end reads of 150 bp as previously reported [33]. Meanwhile, mRNA for each sample was enriched, fragmented and transcribed into cDNA followed by the addition of a single ‘A’ base and subsequent ligation of the adapter. The products were then purified and enriched with PCR amplification. The PCR products were quantified and pooled to make a single strand DNA circle (ssDNA circle), which gave the final library. DNA nanoballs (DNBs) were generated with the ssDNA circle by rolling circle replication (RCR) to enlarge the fluorescent signals at the sequencing process. The DNBs were loaded into the patterned nanoarrays and single-end read of 50 bp were read through on the BGISEQ-500 platform for the following data analysis study.

### 4.6. Raw Data Analysis, Annotation, Mapping and Selection of Differential Expressed Genes (DEGs)

Before any further analysis, raw reads were pre-processed to filter out reads (1) with adaptors, (2) in which unknown bases are more than 5% and (3) that percentage of low-quality reads is larger than 20% [89]. The clean reads were assembled de novo with Trinity pair-end assembly method [90]. The assembled clean reads were clustered to obtain unigenes using the TIGR Gene Indices clustering tools (TGICL) [91]. The clean reads were mapped to the reference transcripts assembled from the de novo transcriptome using Bowtie2. The gene expression levels for each gene were calculated as fragments counts with RSEM software. DEGs between different comparison groups (−6 hAP vs. 0 hAP, 0 hAP vs. 54 hAP, −6 hAP vs. 54 hAP, where a later time point was compared to an earlier one) were determined using DEGseq algorithms with an expression difference threshold of |log_2_Ratio| ≥ 2 and the adjusted *p*-value ≤ 0.001. A local translated nucleotide BLAST (BLASTX) against Arabidopsis protein sequence data set (Araport11, download from TAIR, https://www.arabidopsis.org/index.jsp) was conducted to get the closest homologues in *Arabidopsis* as suggested [92]. Then, each gene in the current study was annotated based on its homologues in *Arabidopsis*. 

### 4.7. Bioinformation Analysis

The common genes among different comparisons were detected by Venny online software (version 2.1, http://bioinfogp.cnb.csic.es/tools/venny/) [93]. The heatmap represents the expression levels of the common genes was conducted in R with package “heatmap” [94], and cluster analysis was made according to similarity of the gene expression under different comparisons. The GO and KEGG analyses were performed as suggested [74] in R with two packages “clusterProfiler” and “pathview” [95,96]. The KEGG pathways were adapted according to the results obtained from R. For the co-expression network, the values of fragments per kilobase per million (FPKM) of genes in all the detected samples were used to calculate the Pearson correlation between each gene pair in R with package “GeneNT” (version 1.4.1) [97] with minor modifications [98]. The correlative relationships among the genes were visualized in Cytoscape (version 3.5.0) [99]. 

### 4.8. Validation of RNA-Seq by qRT-PCR

Nine DEGs were randomly selected to validate the RNA-Seq analysis data, and the gene specific primers are presented in Table S8. For each time point, RNA samples of three biological replicates for RNA-seq were used for quantitative real time PCR validation. The first-strand cDNA was synthesized using a PrimeScript™ RT reagent kit with gDNA Eraser (RR047A, Takara, Japan), based on the manufacturer’s protocol. Three technical replicates were performed on an ABI 7300 Real-Time PCR System (Applied Biosystems, Massachusetts, USA) using TB Green^TM^ Premix Ex Taq^TM^ II (RR820A, TaKaRa, Japan). 60S ribosomal protein was used as the internal control gene. The relative expression levels of the genes were calculated by the 2^−ΔΔ*C*t^ method [100]. 

### 4.9. Statistical Analysis

All the physiological variables were statistically analyzed with one-way ANOVA in SPSS (SPSS Inc., Chicago, IL, USA) and graphs were produced with Microsoft Excel 2016. Tukey’s HSD comparisons were used to determine statistically significant differences among the time points. Differences in the means between time points were considered significant if the *p*-values were less than 0.05. The data distribution was assessed for normality and transformed logarithmically when necessary before performing statistical analysis [101]. The correlation analysis of RNA-seq results between different samples was performed in R with “cor” function.

## Supplementary Materials

Supplementary materials can be found at https://www.mdpi.com/1422-0067/20/15/3707/s1.
