# Supplementary material for: Physiological and Transcriptomic Changes during the Early Phases of Adventitious Root Formation in Mulberry Stem Hardwood Cuttings
Source: Int J Mol Sci. 2019 Jul 29;20(15):3707. doi: 10.3390/ijms20153707 (PMC6696018; doi:10.3390/ijms20153707)
Supplement: Supplementary file 1 [file ijms-20-03707-s001.zip › ijms-547456-supplementary/ijms-547456-supplementary.pdf]

International Journal of
Molecular Sciences

MDPI

Supplementary Materials:

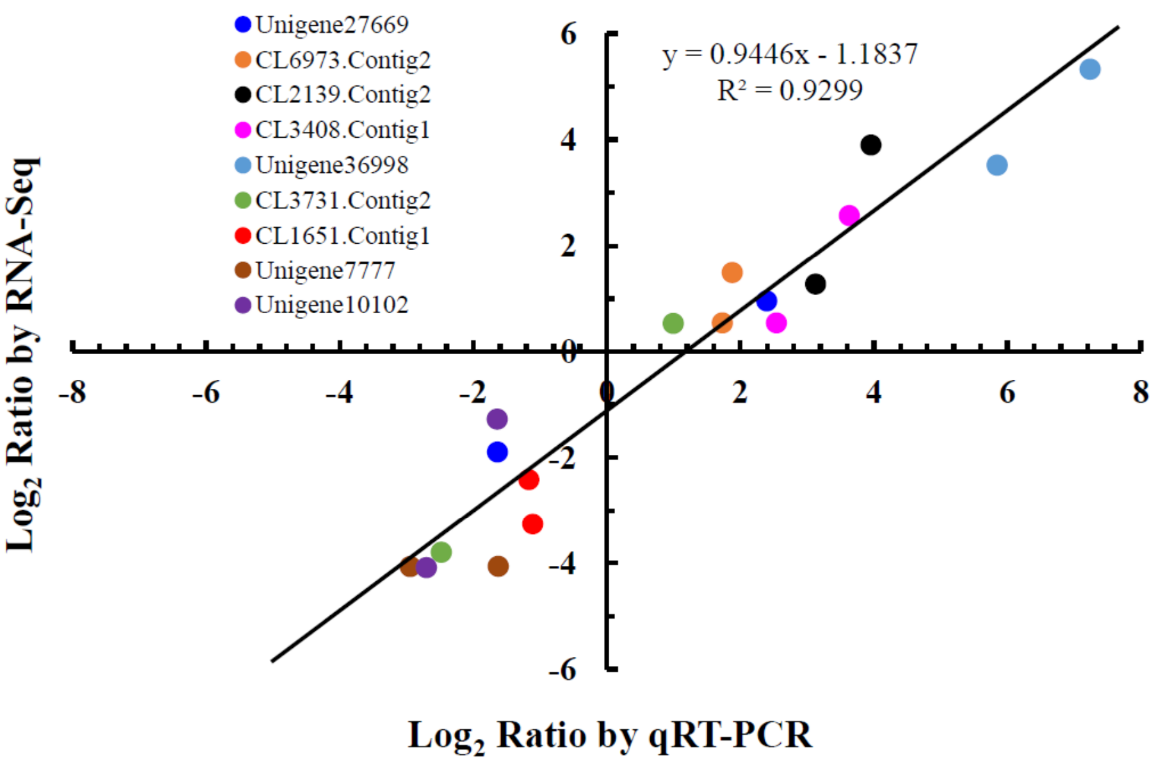

Figure S1. Correlations of RNA-seq results between the samples used in this study. The color code from green to red represents the Pearson values from 0 to 1.

		-6 hAP 1	-6 hAP 2	-6 hAP 3	0 hAP 1	0 hAP 2	0 hAP 3	54hAP 1	54hAP 2	54hAP 3
54 hAP	1	0.49	0.482	0.478	0.575	0.585	0.583	0.995	0.987	1
	2	0.471	0.463	0.46	0.557	0.566	0.564	0.992	1	0.987
	3	0.502	0.494	0.491	0.587	0.597	0.596	1	0.992	0.995
0 hAP	1	0.939	0.933	0.934	0.998	0.998	1	0.596	0.564	0.583
	2	0.943	0.938	0.938	0.998	1	0.998	0.597	0.566	0.585
	3	0.946	0.938	0.94	1	0.998	0.998	0.587	0.557	0.575
-6 hAP	1	0.998	0.998	1	0.94	0.938	0.934	0.491	0.46	0.478
	2	0.995	1	0.998	0.938	0.938	0.933	0.494	0.463	0.482
	3	1	0.995	0.998	0.946	0.943	0.939	0.502	0.471	0.49

pearson value: 1, 0.9, 0.8, 0.6, 0.4, 0

Figure S2. Correlation analysis between RNA-seq results and qRT-PCR analysis of nine DEGs during the early phases of AR formation in mulberry stem hardwood cuttings.

© 2019 by the authors. Submitted for possible open access publication under the terms and conditions of the Creative Commons Attribution (CC BY) license (http://creativecommons.org/licenses/by/4.0/).
